# Early-onset CLL is characterized by enhanced microenvironment-driven metabolic fitness, increased proliferative bias, and accelerated disease progression

**DOI:** 10.3389/fimmu.2026.1936737

**Published:** 2026-09-07

**Authors:** Eugenia Payque, Santiago A. Rodríguez-Zraquia, Marianela Lasagna, Rita Uria, Vanesa A. Guazzone, Victoria Irigoin, Ana Inés Landoni, Victoria Remedi, Virginia Lema, Sabrina Ranero, Gabriela de Galvez, Raúl Gabus, Pablo Muxi, Helga Simon-Molas, Carolina Oliver, Pablo Oppezzo

**Affiliations:** 1 Research Laboratory on Chronic Lymphocytic Leukemia, Institut Pasteur de Montevideo, Montevideo, Uruguay; 2 Immunobiology Academic Unit, School of Medicine, University of the Republic, Montevideo, Uruguay; 3 Facultad de Medicina, Departamento de Histología, Embriología, Biología Celular y Genética, Universidad de Buenos Aires, Buenos Aires, Argentina; 4 Instituto de Investigaciones Biomédicas (INBIOMED), CONICET- Universidad de Buenos Aires, Buenos Aires, Argentina; 5 Hematology Department, Centro de Asistencia del Sindicato Médico del Uruguay (CASMU), Montevideo, Uruguay; 6 Hematology Department, Institución de Asistencia Médica Privada de Profesionales (COSEM), Montevideo, Uruguay; 7 Hospital Maciel, ASSE, Montevideo, Uruguay; 8 Hematology Department, Dirección Nacional de Sanidad de las Fuerzas Armadas, Montevideo, Uruguay; 9 Unidad Académica de Hematología. Hospital de Clínicas, Facultad de Medicina, Universidad de la República, Montevideo, Uruguay; 10 Hematology Department, Hospital Británico, Montevideo, Uruguay; 11 Amsterdam University Medical Centers (UMC) location University of Amsterdam, Experimental Immunology, and location Vrije Universiteit Amsterdam, Hematology, Amsterdam, Netherlands

**Keywords:** CENCAT, CLL - chronic lymphoblastic leukemia, early-onset, metabolic fitness, tumor micoenvironment

## Abstract

Chronic lymphocytic leukemia (CLL) predominantly affects older adults; however, approximately 10% of patients are diagnosed at a younger age. Although overall survival appears comparable across age groups, emerging evidence suggests that early-onset CLL may represent a biologically distinct disease subset. Given the profound effects of aging on immune competence and tumor-host interactions, we investigated whether age at diagnosis influences leukemic cell behavior, microenvironmental responsiveness, and immune dysfunction in CLL. We first analyzed the clinical impact of age at diagnosis in a Uruguayan cohort of 462 CLL patients and subsequently explored the biological basis underlying age-associated differences. Patients were stratified according to age at diagnosis, and disease progression was assessed by time-to-first treatment (TTFT). Metabolic analysis evaluated leukemic cell responses to prototypical tumor microenvironment (TME) stimuli, including CD40L plus IL-4 and CpG-ODN plus IL-15. In parallel, we characterized proliferative and quiescent leukemic fractions in peripheral blood, assessed T-cell exhaustion profiles, and analyzed metabolic reprogramming following microenvironmental stimulation. Patients diagnosed at ≤55 years of age exhibited the shortest TTFT (median 39 months), whereas those aged ≥65 years showed a significantly longer TTFT (median 97,6) confirming the more aggressive clinical course of early-onset CLL, despite the longer overall survival as previously described. Metabolic studies revealed that leukemic cells from younger patients displayed enhanced responsiveness to TME-derived signals, characterized by increased metabolic fitness and greater metabolic reprogramming following stimulation. Moreover, early-onset CLL was enriched in proliferative leukemic fractions, whereas the frequency and distribution of exhausted T-cell subsets were comparable between age groups. Collectively, our findings indicate that age-dependent differences in CLL behavior are primarily associated with intrinsic leukemic cell properties rather than major alterations in T-cell dysfunction. Early-onset CLL is characterized by enhanced metabolic adaptability and enrichment of proliferative leukemic cells, supporting the concept that it constitutes a biologically distinct subset of the disease. Despite the absence of differences in survival between age groups, our results provide new insights into CLL heterogeneity and suggest that age-related differences in leukemic cell–microenvironment interactions may contribute to the more aggressive clinical behavior observed in younger patients.

## Introduction

Chronic lymphocytic leukemia (CLL) is the most common leukemia in adults in Western countries and predominantly affects older individuals, with a median age at diagnosis of approximately 70 years (1). Despite major therapeutic advances, CLL remains incurable and exhibits substantial heterogeneity in clinical behavior and treatment response (2).

The tumor microenvironment (TME) is a central regulator of CLL biology, providing critical survival, proliferative, and anti-apoptotic signals through both cellular interactions and soluble factors (3). Although CLL was historically considered a disease of cellular accumulation, kinetic studies demonstrated that leukemic expansion reflects a dynamic equilibrium between cell proliferation and cell death (4) (5). Consistent with this concept, transcriptomic and single-cell analyses have identified functionally distinct leukemic subpopulations coexisting within individual patients, including quiescent (QF), proliferative (PF), and dividing fractions (DF) (6–9). The relative abundance of these subsets is closely associated with disease evolution, supporting a model in which continuous microenvironmental signaling and dynamic trafficking between peripheral blood (PB) and lymph nodes (LN) enhance leukemic expansion and disease evolution (5, 10).

Microenvironmental signals not only regulate leukemic cell activation but also profoundly reshape cellular metabolism. During circulation between PB and LN, CLL cells undergo marked metabolic adaptations that support proliferation, survival, and therapeutic resistance (11). Increasing evidence indicates that metabolic reprogramming represents a key component of the leukemic response to TME-derived stimuli and contributes to disease aggressiveness (12).

In parallel, CLL is characterized by profound immune dysfunction, manifested by hypogammaglobulinemia, recurrent infections, and autoimmune complications. These alterations are accompanied by extensive remodeling of the T-cell compartment, including expansion of circulating CD8+ T cells and enrichment of CD4+ T cells within lymphoid tissues (13, 14). Furthermore, chronic antigenic stimulation within the leukemic microenvironment promotes T-cell exhaustion, a dysfunctional state associated with impaired antitumor immunity, disease progression, and treatment resistance (15). Together, microenvironment-dependent signaling, metabolic plasticity, and T-cell dysfunction constitute interconnected hallmarks of CLL pathogenesis.

Although CLL is primarily a disease of older adults, approximately 8-10% of patients are diagnosed before the age of 55 years. (16–18). Indeed, younger patients display a higher prevalence of adverse prognostic biomarkers (19) and experience a shorter time-to-first treatment (TTFT) (20), whereas CLL-related and infection-related mortality are more frequent among elderly individuals (21). Despite this earlier disease progression, early-onset patients exhibit an overall survival (O.S.) comparable to that of older patients (17, 22, 23).

Given the central role of TME interactions in CLL pathogenesis, it is plausible that age at disease onset influences the interplay between leukemic cells and their microenvironment, thereby contributing to the biological and clinical differences observed between younger and older patients.

Here, we first validated in a Uruguayan cohort of 462 CLL patients the clinical impact of age at diagnosis on disease progression, as assessed by TTFT and O.S. We then investigated the biological basis that could explain these clinical differences. Specifically, we compared the metabolic responses of CLL cells from young-onset and older patients to typical microenvironmental stimuli, including CD40L plus IL-4 (24) and CpG-ODN plus IL-15 (25). We further characterized the metabolic responses of proliferative and quiescent leukemic fractions from PB and compared their metabolic fitness between young-onset and older CLL patients. Finally, we evaluated T-cell exhaustion profiles in both age groups.

Our results demonstrate that early-onset CLL is characterized by enhanced microenvironment-driven metabolic fitness and enrichment of proliferative leukemic cells, whereas T-cell exhaustion profiles remain unaffected by age at diagnosis. Collectively, these findings reveal previously unrecognized age-dependent biological differences and support the concept that early-onset CLL represents a distinct biological subset with different interactions with the tumor microenvironment, thereby providing new insights into the biological heterogeneity of CLL.

## Methods

### Study design and population

We performed a retrospective, multicenter, observational cohort study within the Uruguayan CLL group (GURU-CLL), drawing on the prospectively maintained registries of six Uruguayan centers including both the public and private healthcare centers: CASMU, COSEM, Hospital Británico, Hospital de Clínicas, Hospital Maciel, and Hospital Militar. Eligible patients had a diagnosis of CLL established according to iwCLL criteria (26). To balance completeness of follow-up with capture of the contemporary treatment era, patients were included if they were diagnosed after 2000 and received first-line therapy after 2010, or if they were diagnosed after 2010 irrespective of treatment status. Patients with monoclonal B-cell lymphocytosis were not included. Of 534 registered patients, 462 had complete data for the analysis of TTFT and constituted the study population. The study was conducted in accordance with the Declaration of Helsinki and approved by the participating institutional review boards.

### Variables and definitions

Analyzed variables included age and sex; healthcare sector (public vs private); Eastern Cooperative Oncology Group (ECOG) performance status; Binet and Rai clinical stage; absolute lymphocyte count (ALC); serum lactate dehydrogenase (LDH) and β_2_-microglobulin; interphase fluorescence *in situ* hybridization (FISH) for del(13q), del(11q), trisomy 12 and del(17p), interpreted using the hierarchical model (27); *TP53* mutation status; and *IGHV* somatic hypermutation status, classified as mutated or unmutated using the conventional 98% germline-identity cutoff (28). Age at diagnosis was categorized *a priori* into three strata: early-onset (≤55 years), 56–64 years, and ≥64 years. The indication for treatment followed iwCLL criteria (26). TTFT was defined as the interval from the date of diagnosis to the start of first CLL-directed therapy; patients who remained untreated were censored at the date of last follow-up.

#### Reagents and antibodies

Key resources and antibodies used for CENCAT analyses and the assessment of T-cell exhaustion-associated molecules are listed in **Table 1**.

**Table 1:** Reagents and antibodies used.

CENCAT Reagents		
L-homopropargylglycine (HPG)	Vector Laboratories	Cat#CCT-1067
THPTA	Vector Laboratories	Cat#CCT-1010
AZDye 488 Azide Plus	Vector Laboratories	Cat#CCT-1475
Oligomycin A	Merck	Cat#75351
2-deoxy-D-glucose (2-DG)	Merck	Cat#D8375
RPMI 1640 medium (with sodium bicarbonate, without methionine, cystine and L-glutamine)	Merck	Cat#R7513
Fetal Bovine Serum, dialyzed, US origin, One Shot™ format	Thermo Fisher	Cat#A3382001
Flow cytometry antibodies		
Cyto-Fast™ Fix/Perm Buffer Set	Biolegend	Cat#426803
CD5-PE-Cy7	Biolegend	Cat: 300622
CD19-PE	Biolegend	Cat: 115508
CD19- Pacific Blue	Biolegend	Cat: 302232
CXCR4-PE	Biolegend	Cat: 306506
α-IgM-PE	Jackson ImmunoResearch	Cat: 709-116-073
α-IgG-AF488	Jackson ImmunoResearch	Cat: 709-546-098
α-CD3-FITC	Biolegend	Cat: 317306
α-CD3-Alex Fluor 700	Biolegend	Cat: 317340
α-CD8-PE-Cy7	Biolegend	Cat: 344750
α-Tim-3-APC	Biolegend	Cat: 345012
α-CD244-Pacific Blue	Biolegend	Cat: 329524
α-TOX-PE	Invitrogen	Cat: 12-6502-82
α-CCR7-Pacific Blue	Biolegend	Cat: 329524
α-CD45RO-APC	Invitrogen	Cat: MHCD45RO05
α-PD1-PerCP/Cyanine5.5	Biolegend	Cat: 621614

#### Metabolic assay: CENCAT

CENCAT (Cellular Energetics through Non-Canonical Amino acid Tagging) is a novel flow cytometry-based technique that uses click chemistry labeling of alkyne-bearing non-canonical amino acids to quantify protein synthesis as a proxy for metabolic activity, based on the principle that cellular energy status is tightly reflected by protein synthesis rate (29). Its compatibility with flow cytometry, low sample requirements, and ability to capture dynamic metabolic responses under physiologically relevant conditions make it a powerful tool for studying metabolic heterogeneity and identifying disease-associated metabolic vulnerabilities. By treating cells with metabolic inhibitors 2-deoxy-D-glucose (2-DG) to inhibit glycolysis, oligomycin to inhibit ATP synthase, or a combination of both, functional metabolic parameters such as: mitochondrial dependence, glucose dependence, and glycolytic capacity can be derived. Of these, mitochondrial dependence, reflecting reliance on oxidative phosphorylation (OXPHOS) activity, and glucose dependence, reflecting the proportion of protein synthesis sustained by glucose oxidation, are reported here as primary readouts.

CENCAT monitors rapid changes in protein translation by detecting the incorporation of the noncanonical amino acid (ncAA) L-homopropargylglycine (HPG) following metabolic pathway inhibition. CENCAT was performed as previously described (30). CLL cells were stimulated with CpG oligodeoxynucleotide (CpG-ODN; 2 µM) plus interleukin-15 (IL-15; 15 ng/mL), or with CD40L (1 µg/mL) plus IL-4 (5 ng/mL) for 24 h in RPMI 1640 medium with 10% heat-inactivated fetal bovine serum (FBS; Gibco, Thermo Fisher Scientific, Cat: 10270-106) at 37 °C, 5% CO_2_. Cells were then depleted of methionine by incubation for 45 min in methionine-free RPMI (Sigma-Aldrich) supplemented with dialyzed FBS (Thermo Fisher Scientific) at 37 °C, 5% CO_2_. Cells were divided and subsequently incubated with control medium (Ctrl), 2-DG 1 mM, oligomycin (10 µM), or a combination of both (2-DG + oligomycin) for 15 min at 37 °C, prior to the addition of HPG (Vector Laboratories) at a final concentration of 200 µM for an additional 30 min. Following HPG incorporation, cells were washed in 1× Dulbecco’s Phosphate Buffered Saline (PBS) and stained with Zombie Violet (1:1000; Thermo Fisher Scientific) for 15 min at room temperature in PBS to assess viability. Cells were then fixed and permeabilized using the Fix/Perm Buffer Set (BioLegend) according to the manufacturer’s instructions.

For the copper(I)-catalyzed azide-alkyne cycloaddition (click) reaction, cells were incubated for 45 min at room temperature in click buffer containing 2.5 mM Cu(II)SO_4_ (Sigma-Aldrich), 10 mM freshly prepared sodium ascorbate (Sigma-Aldrich), 10 mM tris-hydroxypropyltriazolylmethylamine (THPTA; Vector Laboratories), and 0.5 µM AZDye 488 Azide Plus (Vector Laboratories). Cells were subsequently stained with primary antibodies against surface markers CD5-PE-Cy7 and CD19-PE for 30 min at 4 °C. For some experiments CXCR4-PE and CD19-PB antibodies were used. Data were acquired on an Attune NxT flow cytometer (Thermo Fisher Scientific). Unstained and FMO controls were included for each culture condition to allow autofluorescence subtraction.

Mean fluorescence intensity (MFI) values were extracted from the population of interest. Relative cellular reliance on glucose metabolism and mitochondrial respiration was assessed by comparing the MFIs of ncAA labeling under different inhibitor conditions, as follows:


Mitochondrial dependence (%) = [(Control−Oligomycin)/(Control− 2DG+Oligomycin)]×100

Glucose dependence(%)=[(Control−2DG)/(Control−2DG+Oligomycin)]×100

Glycolytic capacity(%)=100−[(Control−Oligomycin)/(Control−2DG+Oligomycin)]×100

### Flow cytometry analysis for T-cells

Following thawing, PBMCs (1 × 10^6^ cells per well) were seeded into 96-well plates and washed twice with 1× PBS without calcium and magnesium (Invitrogen™, Cat: 14190-144) supplemented with 5% FBS. Surface staining was performed by incubating the cells with the appropriate antibody cocktail (**Table 1**) for 30 min at 4 °C. Cells were subsequently washed twice and fixed with 2% paraformaldehyde for 15 min at room temperature in the dark. After two additional washes, cells were resuspended in PBS containing 5% FBS and acquired by flow cytometry.

For intracellular staining, fixed cells were washed twice with 1× permeabilization buffer (Cyto-Fast Perm/Wash, BioLegend, Cat: 750000135) and incubated overnight at 4 °C with the corresponding intracellular antibody cocktail (**Table 1**). Cells were then washed twice with permeabilization buffer, followed by two washes with PBS containing 5% FBS, resuspended in the same buffer, and analyzed by flow cytometry. Data were acquired with Attune NxT flow cytometer (Thermo Fisher Scientific) and Cytek^®^ Aurora and analyzed by FlowJo 10.8 version.

### Statistical analysis

Categorical variables were summarized as frequencies and percentages, whereas continuous variables were expressed as medians and ranges. Baseline characteristics were compared across age strata using the chi-square test or Fisher’s exact test for categorical variables and the Kruskal–Wallis test for continuous variables. Additional statistical analyses, including unpaired and paired *t*-tests, one-way ANOVA, Mann–Whitney tests, and Wilcoxon matched-pairs tests, were performed as indicated in the corresponding figure legends. TTFT and O.S. were estimated using the Kaplan–Meier method, and differences between age groups were assessed using the log-rank test. All statistical tests were two-sided, and *P* values <0.05 were considered statistically significant. Statistical analyses were performed using SPSS (IBM Corp., Armonk, NY, USA) and GraphPad Prism (GraphPad Software, San Diego, CA, USA).

## Results

### Patients with early-onset CLL require treatment earlier than their older counterparts

Among 462 evaluable patients, 208 needed first line treatment. Men 295 and 167 women. Median age was 68 years old (33-94). Typical cytogenetic aberrations were observed in 59% of cases. IGHV mutational profile was 50% mutated, clinical Binet stage: A = 66%, B = 20% and C = 14%. Public system: 259 (53,4%), private: 226 (46,6%). The median follows up: 62 months (0,2-295).

**Table 2** shows the characteristic of the patients according to the age group. Young patients (≤55 years) accounted for 17%. There are not differences in sex, LDH, B2 microglobulin, citogenetics, mutational IGVH status or initial management. However, we found differences in ECOG status, with elderly patients being less fit, and in stage at diagnosis. Younger patients more frequently presented with advanced Binet or RAI stages and higher lymphocyte counts. They also exhibited a higher proportion of TP53 alterations; however, the limited number of cases precludes definitive conclusions regarding this association.

**Table 2:** Baseline characteristics of the GURU-CLL cohort according to age at diagnosis.

Characteristic	≤55 y (n=79)	56–64 y (n=105)	≥65 y (n=278)	p
Median age, y (range)	52 (33–55)	61 (56–64)	74 (65–94)	—
Sex, n (%)				0.472
Female	28 (35.4)	33 (31.4)	106 (38.1)	
Male	51 (64.6)	72 (68.6)	172 (61.9)	
ECOG PS, n (%)^a^				0.0001
0–1	60 (98.4)	83 (98.8)	176 (84.6)	
2–4	1 (1.6)	1 (1.2)	32 (15.5)	
Binet stage, n (%)^b^				0.043
A	43 (55.1)	70 (70)	191 (72.1)	
B	22 (28.2)	18 (18)	40 (15.1)	
C	13 (16.7)	12 (12)	34 (12.8)	
Rai stage, n (%)^c^				0.026
0	23 (37.7)	45 (49.5)	121 (50)	
I	12 (19.7)	22 (24.2)	45 (26.4)	
II	15 (24.6)	13 (14.3)	31 (12.8)	
III	7 (11.5)	6 (6.6)	15 (6.2)	
IV	4 (66)	5 (5.5)	11 (4.5)	
Median ALC, ×10^9^/L	54.5	45.8	32.1	0.039
Elevated LDH, n (%)	12 (17.4)	17 (16.2)	45 (14.4)	0.290
Elevated β_2_-microglobulin, n (%)	18 (26)	40 (38)	99 (36)	0.703
FISH/cytogenetics, n (%)				NS
del(13q)	7 (8.9)	6 (5.7)	10 (3.6)	
del(11q)	4 (5.1)	5 (4.8)	8 (2.9)	
Trisomy 12	5 (6.3)	4 (3.8)	5 (1.8)	
del(17p)/TP53	3 (3.8)	2 (1.9)	5 (1.8)	
Normal	16 (20.3)	9 (8.6)	21 (7.9)	
Not performed	44 (55.7)	79 (75.2)	228 (82)	
TP53 mutated, n (%)	6 (23)	3 (2)	4 (1.4)	0.04
IGHV status, n (%)^d^				NS
Mutated	27 (34)	23 (22)	42 (15)	
Unmutated	26 (33)	24 (23)	43 (15)	
Initial management, n (%)				0.003
Watch and wait	32 (40.5)	56 (53.3)	166 (59.7)	
Frontline treatment	47 (59.5)	49 (46.7)	112 (40.3)	

ALC, absolute lymphocyte count; ECOG PS, Eastern Cooperative Oncology Group performance status; FISH, fluorescence *in situ* hybridization; IGHV, immunoglobulin heavy-chain variable region gene; LDH, lactate dehydrogenase; NS, not significant. Percentages are calculated within each age group from patients with available data. ^a^Data available for 355 patients; ^v^452 patients; ^c^414 patients; ^d^IGHV status assessed in 109 patients. Statistical comparisons: chi-square/Fisher exact test (categorical) and Kruskal–Wallis test (continuous).

**Figure 1:**
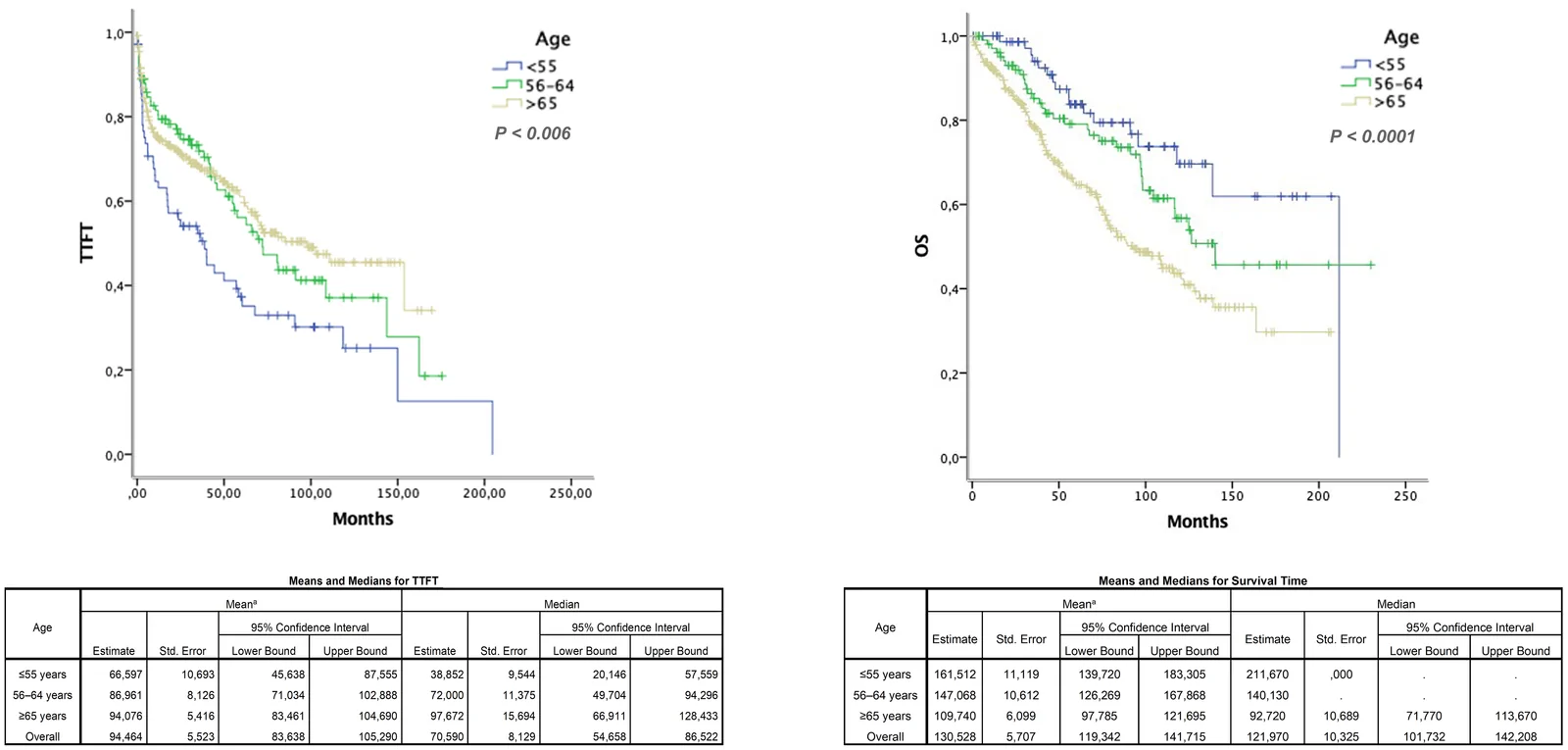
Time to first treatment (TTFT) and O.S. according to age at diagnosis (≤50, 51–65, and ≥66 years). TTFT and O.S. were estimated with Kaplan-Meier and comparations were perform with log rank test. p<0.05, ns: not significant.

Patients aged ≤55 years (n = 79) exhibited the shortest TTFT, with a median of 39 months (95% CI, 20–57), compared with 72 months (95% CI, 49–94) and (97,6 months; 95% CI, 67–128) in patients aged 56–64 years (n = 105) and ≥65 years (n = 278), respectively. The median TTFT for the entire cohort was 70.6 months (95% CI, 54.6–86.5), and differed significantly among age groups (p = 0.006; **Figure 1A**). Furthermore, consistent with previous reports (17, 19) patients aged ≤55 years exhibited significantly longer overall survival despite their shorter TTFT. Median overall survival was 211 months in the early-onset group compared with 92 months in patients diagnosed at older ages (p = 0.0001; **Figure 1B**).

Thus, analysis of this real-world Uruguayan cohort demonstrates that patients with early-onset CLL (≤55 years) require treatment substantially earlier than older-onset patients (≥65 years), a phenomenon that has been associated with more advanced disease stage and higher lymphocyte counts at diagnosis.

### CENCAT confirms metabolic reprogramming of CLL cells and reveals distinct metabolic responses to CD40L+IL-4 and CpG-ODN+IL-15 stimulation

Previous studies have shown that CD40-mediated B-cell activation promotes preferential glucose flux through the pentose phosphate pathway and increased glutaminolysis to sustain OXPHOS activity (12, 31, 32). In contrast, T-independent signals emulated by CpG/TLR9 activation mostly enhanced glycolysis in naïve B cells (33).

Specifically, in CLL, metabolic responses have been mainly investigated following BCR and/or CD40 stimulation, both of which increase mitochondrial respiratory capacity, glycolytic activity, glucose and glutamine utilization (12, 34). In contrast, the metabolic consequences of TLR9 activation and the relative contribution of T-dependent versus T-independent signaling pathways remain poorly characterized.

**Figure 2:**
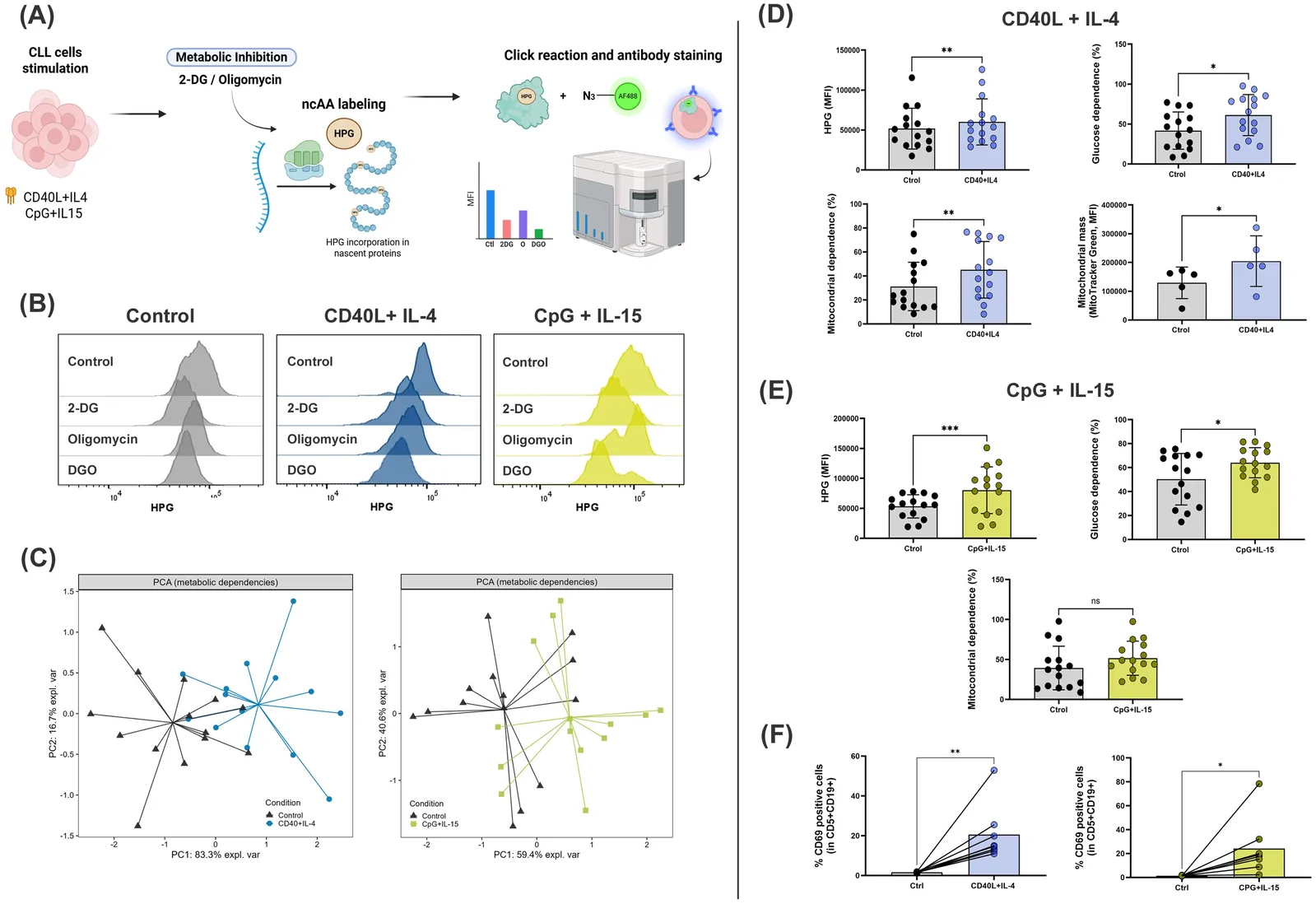
CENCAT-based metabolic profiling of CLL cells following TME-like stimulation. **(A)** PBMCs isolated from CLL patients (n = x) were stimulated with CD40L (1 µg/mL) + IL-4 (5 ng/mL), CpG-ODN (2 µM) + IL-15 (15 ng/mL), or left unstimulated (Control), after which CLL cells (CD5^+^/CD19^+^) were subjected to CENCAT analysis. Created with BioRender.com. **(B)** Representative histograms of HPG incorporation under Control, 2-DG, oligomycin, and 2-DG+oligomycin conditions in unstimulated, CD40+IL-4-, and CpG-ODN+IL-15-stimulated CLL cells (CD5+CD19+). **(C)** Multilevel PCA score plot (multilevel factor = patient) based on mitochondrial and glucose dependence of CLL cells following CD40+IL-4 and CpG-ODN+IL-15 stimulation. Control samples are depicted in black (triangles), CD40+IL-4-stimulated samples in blue (circles), and CpG-ODN+IL-15-stimulated samples in yellow-green (squares). Lines connect each individual data point to the group centroid. Analysis was performed using the PCA function of the mixOmics package in R (52). **(D, E)** HPG incorporation (MFI), mitochondrial dependence (%), and glucose dependence (%) of CLL cells (CD5+CD19+) under Control, CD40+IL-4, and CpG-ODN+IL-15 conditions (n = 15). Mitochondrial mass assessed by MitoTracker Green staining in CLL cells (CD5+CD19+) following CD40+IL-4 stimulation compared to unstimulated controls (n = 6). **(F)** CD69 surface expression on CLL cells (CD5+CD19+) following CD40+IL-4 and CpG-ODN+IL-15 stimulation compared to unstimulated controls (n = 8). Data are shown as mean ± SD. Statistical analysis was performed using a paired two-tailed Student’s t-test. *p<0.05, **p<0.01, ***p<0.001, ns: not significant.

To address this question, we employed CENCAT, a recently developed flow cytometry-based approach that simultaneously assesses cellular energetic reprogramming and protein synthesis at the single-cell level (29). A schematic representation of the assay and the metabolic profiles of CLL cells induced by CD40L+IL-4 and CpG-ODN+IL-15 stimulation are shown in **Figures 2A–C**.

Representative histograms demonstrated a progressive reduction in HPG incorporation following metabolic inhibition with 2-deoxy-D-glucose (2-DG) to inhibit glycolysis, oligomycin to inhibit ATP synthase, or a combination of both, under unstimulated, CD40L+IL-4, and CpG-ODN+IL-15 conditions, validating the robustness of the CENCAT readout in our experimental setting (**Figure 2B**). To obtain an integrated view of stimulus-induced metabolic reprogramming, principal component analysis (PCA) was performed using all CENCAT-derived parameters (**Figure 2C**). CD40L+IL-4 stimulation induced a marked separation from unstimulated controls, with PC1 accounting for 83.3% of the within-patient variance, consistent with a highly coordinated metabolic response. In contrast, CpG-ODN+IL-15 induced a less pronounced separation (PC1 = 59.4%, PC2 = 40.6%), indicating greater inter-patient variability and suggesting distinct mechanisms of metabolic reprogramming.

Analysis of individual metabolic parameters further supported these differences (**Figure 2D**). CD40L+IL-4 significantly increased both mitochondrial dependence and glucose dependence, indicating coordinated enhancement of OXPHOS and glucose utilization.To further characterize the mitochondrial response, MitoTracker Green staining was performed in a subset of patients (n = 5) and revealed a significant increase in mitochondrial mass following CD40+IL-4 stimulation, indicating that the increase in mitochondrial dependence observed by CENCAT is accompanied by mitochondrial biogenesis (**Figure 2D**). In contrast, although CpG-ODN+IL-15 significantly increased protein synthesis and glucose dependence, mitochondrial dependence remained unchanged relative to unstimulated controls (**Figure 2E**). As activation control of successful cellular activation, CD69 surface expression was assessed following both stimuli (CD40+IL-4 and CpG-ODN+IL-15) compared to unstimulated controls (**Figure 2F**).

Together, these findings indicate that CD40+IL-4 and CpG-ODN+IL-15 activate CLL cell metabolism through partially distinct mechanisms both increasing overall metabolic activity and glucose dependence, but diverging in their effect on mitochondrial reliance.

### Enhanced CD40L+IL-4-induced metabolic reprogramming, but not CPG-ODN+IL-15 responsiveness, distinguishes early-onset from older CLL

The shorter TTFT observed in early-onset CLL (≤55 years), both in previous studies (22) and in our Uruguayan cohort, suggests the existence of biological differences that may contribute to its more aggressive clinical behavior. Given the central role of metabolic adaptation in leukemic cell activation and proliferation, we investigated whether age at diagnosis influences the metabolic responsiveness of CLL cells to microenvironmental stimulation.

To this end, metabolic reprogramming was assessed by CENCAT following stimulation with CD40L+IL-4 or CpG-ODN+IL-15 in younger (≤55 years) and older (≥55 years) CLL patients.

**Figure 3:**
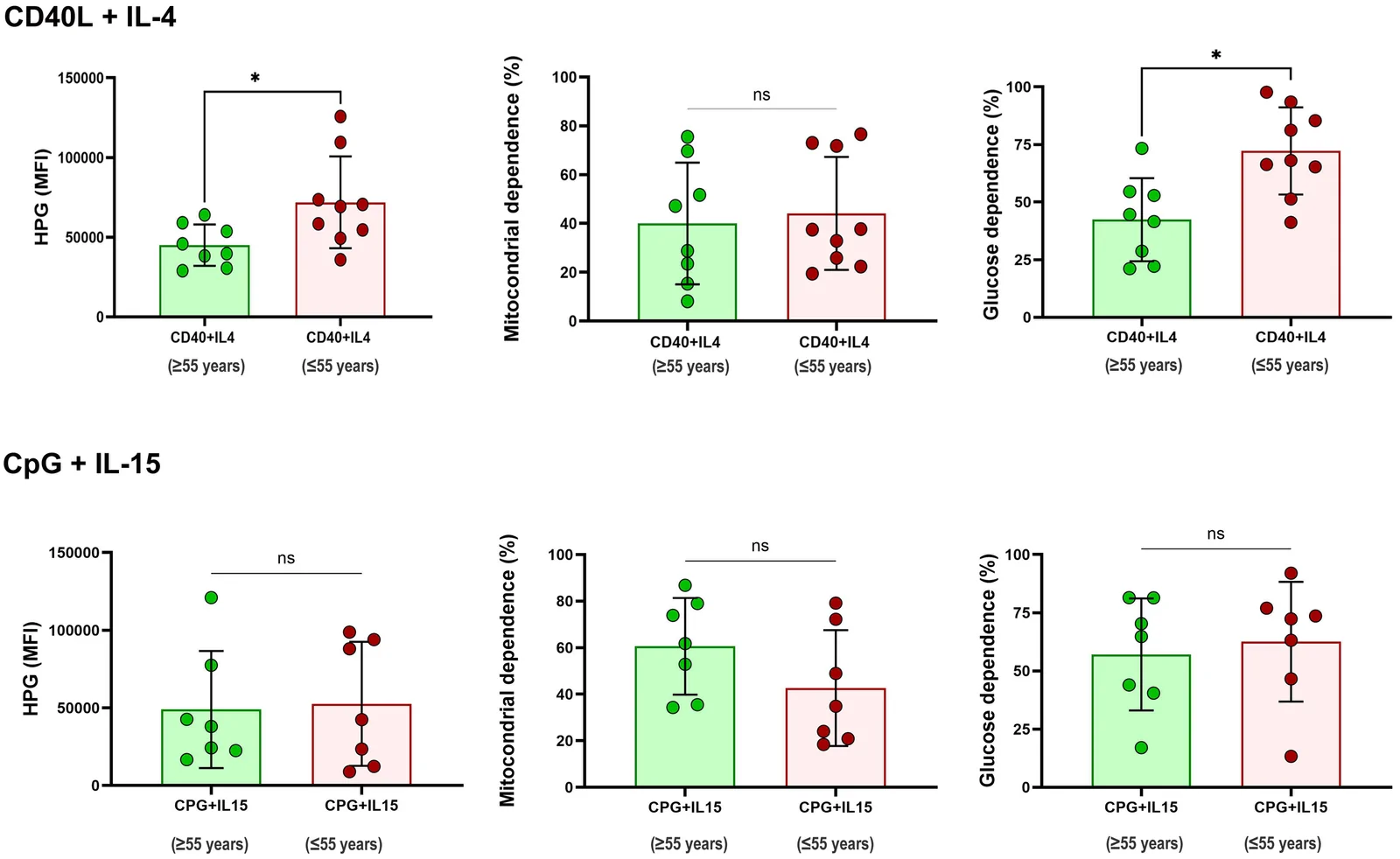
Younger CLL patients exhibit greater metabolic activation in response to CD40+IL-4 but not CpG-ODN+IL-15 stimulation as measured by CENCAT. HPG incorporation (MFI), mitochondrial dependence (%), and glucose dependence (%) assessed by CENACT of CLL cells (CD5+CD19+) from older (>55 years) and younger (≤55 years) CLL patients stimulated with CD40L (1 µg/mL) + IL-4 (5 ng/mL) (upper panels) or CpG-ODN (2 µM) + IL-15 (15 ng/mL) (lower panels). Data are shown as mean ± SD. Statistical analysis was performed using an unpaired two-tailed Student’s t-test. *p<0.05, ns: not significant.

Following CD40L+IL-4 stimulation, CLL cells from younger patients displayed significantly higher protein synthesis and glucose dependence than those from older patients, as reflected by increased HPG incorporation (mean MFI: 69,291 vs. 42,715, respectively; *p* = 0.0274) and glucose dependence (68.1% vs. 43.1%, respectively; *p* = 0.0152; *n* = 17) (**Figure 3**). In contrast, mitochondrial dependence did not differ significantly between age groups. Likewise, no age-associated differences were observed following CpG-ODN+IL-15 stimulation, indicating that the enhanced metabolic responsiveness of early-onset CLL is selective for CD40-mediated activation.

These findings identify age-dependent differences in the metabolic reprogramming of CLL cells and demonstrate that early-onset CLL exhibits an enhanced response to CD40L+IL-4 stimulation, characterized by increased protein synthesis and glucose utilization. Notably, these differences were not accompanied by changes in mitochondrial dependence, suggesting that the metabolic advantage of early-onset CLL is primarily driven by glucose metabolism.

Collectively, our results support the notion that heightened responsiveness to specific microenvironmental signals may contribute to the accelerated clinical course observed in younger patients.

### Early-onset CLL is enriched in proliferative fractions exhibiting enhanced microenvironment-driven metabolic fitness

Given the enhanced glucose-dependent metabolic response to CD40L+IL-4 stimulation observed in early-onset CLL, and considering previous reports showing that proliferative CLL fractions exhibit increased glucose uptake and mitochondrial mass compared with quiescent peripheral blood leukemic cells (34), we next investigated whether age-associated differences in metabolic fitness were linked to changes in the distribution and metabolic properties of distinct leukemic subpopulations. Specifically, we assessed the frequency of proliferative (PF) and quiescent (QF) fractions and further characterized the metabolic profiles of PF, QF, and the double-positive fraction (DF), defined according to CXCR4 and CD5 surface expression as previously described (6, 9), in early-onset CLL patients.

**Figure 4:**
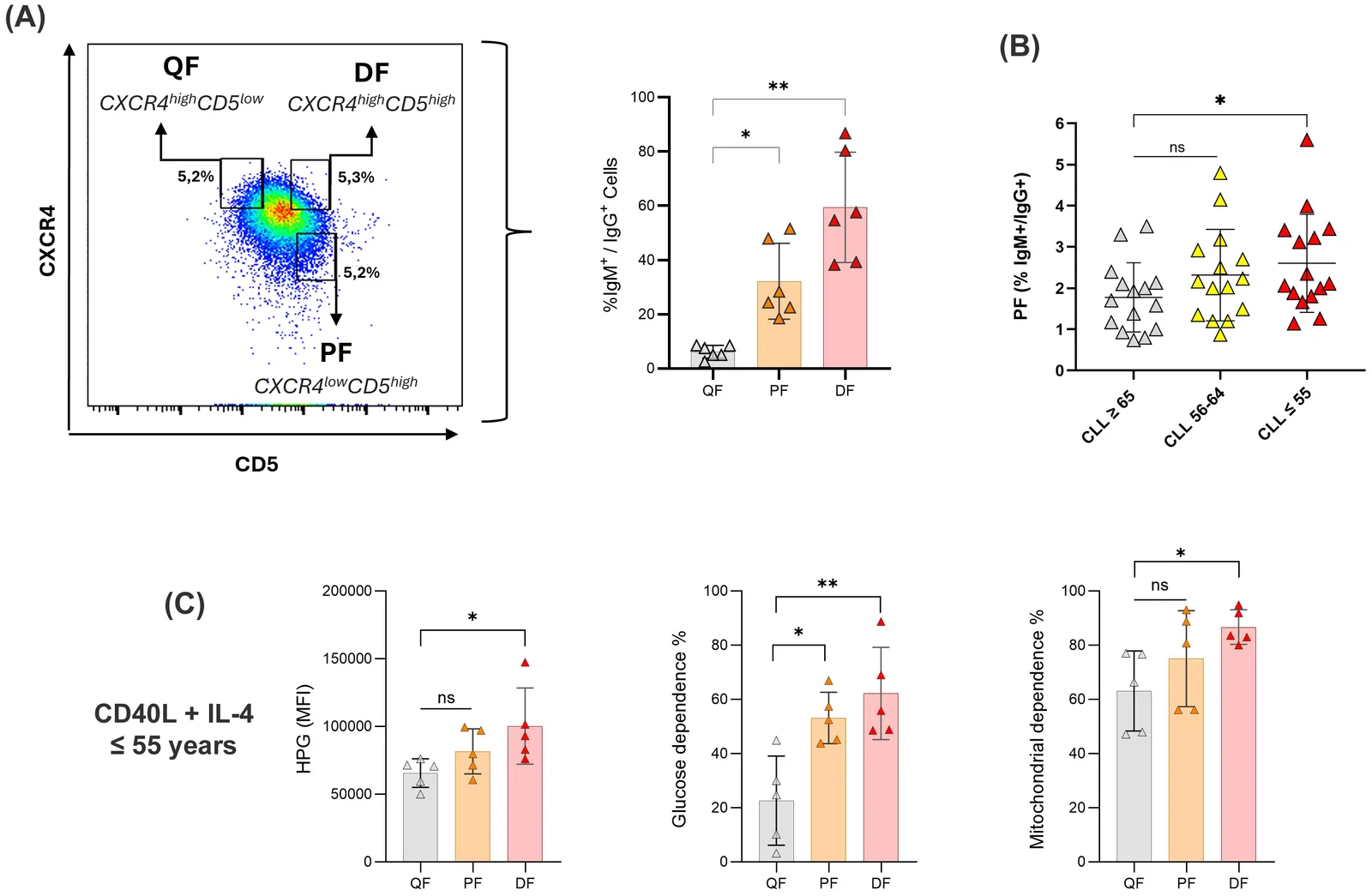
Younger CLL patients show a higher proportion of proliferating cells with enhanced metabolic activity. **(A)** Gating strategy for the identification of quiescent (QF, CXCR4^bright^CD5^dim^), proliferating (PF, CXCR4^dim^/CD5^bright^), and dividing (DF, CXCR4^bright^/CD5^bright^) CLL intraclonal fractions by flow cytometry (left). The proportion of IgM+/IgG+ cells within each fraction was assessed (right, n = 6). Data are shown as mean ± SD. Statistical analysis was performed using one-way ANOVA. **p<0.01. **(B)** Proportion of proliferating fraction (PF, % IgM+/IgG+) in CLL patients stratified by age at diagnosis: older (CLL >65 years), intermediate (CLL 56–64 years), and younger (CLL ≤55 years) (n = 15 per group). Data are shown as mean ± SD. Statistical analysis was performed using an unpaired two-tailed Student’s t-test. *p<0.05, ns: not significant. **(C)** HPG incorporation (MFI), glucose dependence (%), and mitochondrial dependence (%) assessed by CENCAT across QF, PF, and DF fractions from younger (≤55 years) CLL patients stimulated with CD40L (1 µg/mL) + IL-4 (5 ng/mL) for 24 h (n = 5). Data are shown as mean ± SD. Statistical analysis was performed using repeated measures one-way ANOVA with *post-hoc* multiple comparisons test. *p<0.05, **p<0.01, ns: not significant.

Previous studies from our laboratory identified a proliferative CLL fraction characterized by ongoing class-switch recombination (IgM^pos^/IgG^pos^) (10), which provides an alternative approach to identify proliferative leukemic cells. Since this subset shares key biological features with the CXCR4^dim^/CD5^bright^ proliferative fraction described by the Chiorazzi laboratory (6), including activation-induced cytidine deaminase (AID) expression (35) and sensitivity to ibrutinib (36), we first confirmed the overlap between both populations. As shown in **Figure 4A**, the majority of IgM^pos^/IgG^pos^ cells co-localized with both the CXCR4^dim^/CD5^bright^ (PF) and the CXCR4^brigth^/CD5^bright^ (DF), whereas minimal overlap was observed with the quiescent CXCR4^bright^/CD5^dim^ fraction (QF vs PF; mean=32.17, CI -45,92 to -6,055; p = 0,0176 and QF vs DF; mean = 59.42, -83,12 to -23,35: p= 0,0045, n=6, One -way ANOVA test). These findings confirm that both approaches identify a common proliferative/dividing leukemic compartment.

We next quantified the frequency of PF and QF cells according to age at diagnosis. Early-onset CLL patients (≤55 years) exhibited a significantly higher proportion of proliferative leukemic cells than older patients (≥65 years) (mean PF: 2.60% ± 0.83 vs. 1.77% ± 0.38, respectively; *n* = 30, *p* = 0.0369, unpaired two-tailed *t*-test; **Figure 4B**).

To determine whether this expanded proliferative compartment was associated with enhanced metabolic activity, distinct leukemic fractions were identified by gating based on CXCR4 and CD5 surface expression from PB samples of early-onset CLL patients (≤55 years, *n* = 5), (**Figure 4A**) and analyzed by CENCAT. Metabolic profiling revealed marked heterogeneity among intraclonal leukemic fractions (**Figure 4C**). Following CD40L+IL-4 stimulation, HPG incorporation was significantly higher in the DF than in the QF (mean MFI: 34,694 vs. 26,651, respectively; *P* = 0.0436), indicating increased protein synthesis in actively dividing leukemic cells. Similarly, glucose dependence was significantly increased in the DF compared with the QF (mean difference: 39.58%; 95% CI, 23.01–56.15; *p* = 0.0027), consistent with enhanced glucose utilization. Furthermore, mitochondrial dependence was also significantly higher in the DF than in the QF (mean difference: 23.63%; 95% CI, 7.97–39.29; *p* = 0.0138), demonstrating increased reliance on mitochondrial metabolism. Collectively, these findings reveal a progressive increase in metabolic activity along the QF→PF→DF continuum.

Together with the enhanced metabolic response to CD40L+IL-4 observed in early-onset CLL (**Figure 3**), these results suggest that the more aggressive clinical behavior of younger patients is associated with an expanded proliferative compartment exhibiting heightened metabolic reprogramming and increased energetic demands supported by both glucose utilization and mitochondrial metabolism.

### T-cell exhaustion profiles are largely preserved across early-onset and older CLL patients

**Figure 5:**
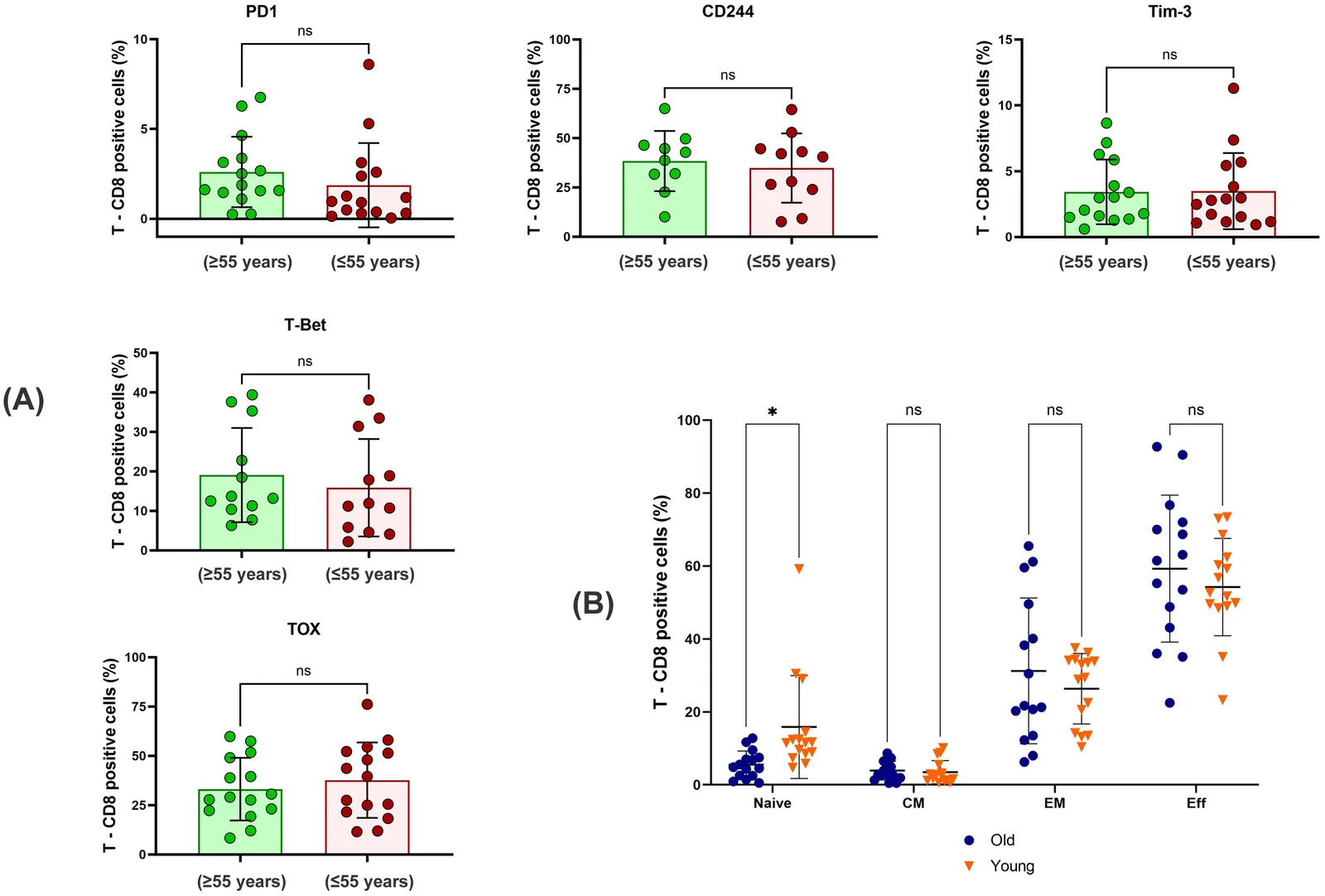
Flow cytometry assessment of exhaustion-associated markers and T-cell profiles in T CD8^+^ cells. **(A)** Frequency of T cells expressing the inhibitory receptors PD-1, CD244, and TIM-3, as well as the transcription factor TOX, all associated with T-cell exhaustion. Results are expressed as the percentage of positive cells. T-bet was included as an additional marker to assess effector polarization. **(B)** Distribution of naive (CD45RO⁻CCR7^+^), central memory (CM, CD45RO^+^CCR7^+^), effector memory (EM, CD45RO^+^CCR7⁻), and effector (Eff, CD45RO⁻CCR7⁻) CD8^+^ T-cell subsets across the study groups. Statistical significance was determined using an unpaired t-test. Data are presented as mean ± SD. *P < 0.05.

To determine whether age at diagnosis influences the differentiation and exhaustion status of T cells in CLL, we compared younger (≤55 years, *n* = 12–15) and older (≥65 years, *n* = 12–15) patients by multiparametric flow cytometry. Despite the distinct clinical behavior of these subgroups and the age-associated differences observed in leukemic cell metabolic responsiveness, T-cell exhaustion profiles were largely comparable between groups. Specifically, no significant differences were detected in the percentage of CD8+ T-cells expressing the inhibitory receptors PD-1, CD244, and TIM-3, nor in the expression of the transcription factors TOX and T-BET, which are involved int eh regulation of T-cell exhaustion and effector differentiation, respectively (**Figure 5A**).

We next evaluated the distribution of CD8+ T-cell differentiation subsets. The frequencies of effector (EF), effector memory (EM), and central memory (CM) CD8+ T cells were comparable between younger and older patients. In contrast, a significant difference was observed within the immature compartment, with younger patients displaying a higher proportion of naïve CD8+ T cells than older individuals (**Figure 5B**).

Collectively, these findings indicate that, despite marked differences in leukemic cell metabolic fitness and proliferative capacity, age at disease onset has a limited impact on the overall exhaustion and differentiation profile of CD8+ T cells in CLL. The higher frequency of naïve CD8+ T cells observed in younger patients is consistent with age-related changes in immune composition but does not appear to translate into major differences in T-cell exhaustion.

## Discussion

A hallmark of adaptive immunity is the ability of lymphocytes to rapidly transition from quiescence to activation following antigen encounter. In B cells, B-cell receptor (BCR) engagement alone is insufficient to sustain a fully functional response, requiring additional co-stimulatory signals and cytokines to support activation, proliferation, and differentiation (37). These processes are accompanied by extensive transcriptional reprogramming and metabolic reprogramming, enabling cells to meet the increased bioenergetic and biosynthetic demands associated with immune activation (12). While BCR stimulation rapidly enhances both glycolysis and OXPHOS, sustained metabolic reprogramming depends on complementary signals delivered through CD40 and TLR9 engagement, as well as microenvironment-derived factors such as BAFF, which collectively preserve glycolytic capacity and mitochondrial function (37, 38).

In CLL, this microenvironment-driven metabolic plasticity is particularly relevant, as proliferation and survival predominantly occur within secondary lymphoid organs, where specialized microenvironmental niches provide the signals required for leukemic cell activation and expansion (39). Following stimulation in lymphoid tissues, CLL cells recirculate into the PB, where they largely adopt a quiescent state characterized by predominant reliance on mitochondrial respiration and OXPHOS to meet basal energetic requirements until they either re-enter tissue niches and receive renewed stimulation or undergo apoptosis (12). Upon exposure to microenvironmental signals such as CD40L and IL-4, CLL cells undergo extensive metabolic remodeling characterized by coordinated upregulation of both OXPHOS and glycolysis, alongside increased utilization of glucose and glutamine to support the heightened biosynthetic and energetic demands of cellular proliferation (11, 34). Consistently, proliferative CLL cells residing in secondary lymphoid organs and the small proliferating fraction detectable in PB retain this metabolically active phenotype, characterized by increased glucose uptake and upregulation of glucose transporters and glycolytic enzymes. Despite this well-established metabolic heterogeneity, whether it differs according to patient characteristics such as age at diagnosis remains an open question (11).

According to the National Cancer Institute’s Surveillance, Epidemiology, and End Results (SEER) database, approximately 11% of patients diagnosed with CLL are younger than 55 years of age (40). Similar studies conducted in Europe have reported frequencies ranging from 7% to 20% (19, 23, 41) highlighting early-onset CLL as a clinically relevant subgroup with distinct biological and clinical features.

In the present study, we evaluated the incidence and clinical characteristics of early-onset CLL in a Uruguayan cohort of 462 patients. We identified 79 patients diagnosed at ≤55 years of age (17%), a frequency comparable to that reported in European cohorts, possibly reflecting the strong European ancestry of the Uruguayan population. Consistent with previous reports, younger patients exhibited a significantly shorter TTFT while maintaining a superior OS compared with patients diagnosed at older ages, supporting the notion that early-onset CLL follows a distinct clinical course.

To gain biological insights into the distinct clinical behavior of young- and older-onset CLL, we investigated the metabolic programs induced by TME stimulation using CENCAT. As an initial step, we validated this approach in 15 CLL samples irrespective of patient age.

CD40+IL-4 stimulation induced a metabolic profile consistent with that previously characterized in CLL cells by extracellular flux analysis using the Seahorse XF analyzer, the current gold standard for metabolic profiling, including enhanced basal and maximal oxygen consumption rate, spare respiratory capacity, and basal extracellular acidification rate, reflecting the coordinated upregulation of both OXPHOS and glycolysis together with broader anabolic and catabolic pathway activation (34). Consistent with these observations, flow cytometry-based assessment of glucose uptake and mitochondrial mass further confirmed enhanced metabolic activity in CD40-stimulated CLL cells (34), findings that were corroborated in the present study by the significant increase in mitochondrial mass observed by MitoTracker Green staining following CD40+IL-4 stimulation. Importantly, our results demonstrate that CENCAT effectively captures these microenvironment-driven metabolic adaptations, supporting its utility as a flow cytometry-based platform for metabolic profiling in CLL.

In contrast, the metabolic consequences of CpG-ODN+IL-15 stimulation have not been previously characterized in CLL cells. Nevertheless, Ueno et al. reported that TLR9 engagement rapidly enhances glycolysis in normal naïve B cells within 30 minutes of stimulation (33). Consistent with these observations, CpG-ODN+IL-15 stimulation in our cohort induced a marked increase in protein synthesis and glucose dependence, indicating greater reliance on glucose as an energy source. This metabolic reprogramming was not accompanied by increased mitochondrial dependence compared with unstimulated cells, suggesting that TLR9-driven activation in CLL engages a predominantly glucose-dependent metabolic program without substantially remodeling mitochondrial metabolism. Together, these findings extend previous observations in normal B cells and provide the first evidence that TLR9 stimulation promotes a predominantly glucose-driven metabolic program in CLL cells.

Although relatively uncommon, early-onset CLL carries a disproportionate clinical burden. Younger patients face the longest cumulative exposure to the disease and experience a significant reduction in life expectancy compared with age- and sex-matched individuals from the general population, despite their longer absolute survival relative to older patients. Seeking biological explanations for these clinical differences, we compared the metabolic responses of CLL cells from young- and older-onset patients following TME stimulation. Our results revealed that CLL cells from younger patients displayed significantly higher protein synthesis and glucose dependence following CD40+IL4 stimulation, whereas mitochondrial dependence remained comparable between age groups.

These findings are consistent with previous studies showing that CLL cells from high-risk patients exhibit enhanced glycolytic activity compared with those from low-risk cases (42), suggesting that increased glucose utilization may represent a common feature of biologically aggressive disease. In support of this notion, PF cells recently egressed from LN display increased glucose uptake together with elevated expression of glucose transporters and glycolytic enzymes (11, 34). A similar metabolic phenotype has also been reported in CLL cells engaged in close interactions with stromal cells within the tumor microenvironment (43).

Collectively, these observations suggest that CLL cells from early-onset patients are metabolically primed toward a more activated and proliferative state, resembling the metabolic profile associated with aggressive disease biology. Supporting this, we observed a significant expansion of the PF undergoing ongoing class-switch recombination in early-onset CLL compared with patients aged ≥65 years, indicating increased proliferative activity in this subgroup.

Motivated by these findings, we next applied CENCAT to characterize the metabolic responses of the distinct CXCR4/CD5-defined CLL subpopulations from patients aged ≤55 years following CD40L/IL-4 stimulation.

These fractions included the quiescent fraction QF, (CXCR4^bright^/CD5^dim^), composed of older cells poised to re-enter lymphoid tissues; the PF, (CXCR4^dim^/CD5^bright^), enriched in recently divided cells in G1-phase that have egressed from lymph nodes (6) and the dividing fraction DF, (CXCR4^bright^/CD5^bright^), recently described as a distinct population enriched in actively cycling cells in S/G2/M phase, expressing high levels of IgM together with the LN homing receptors CCR7 and CXCR5, and expanding at the time of clinical relapse (9). Notably, CENCAT results revealed a progressive increase in metabolic activity along the QF→PF→DF axis, with the highest metabolic responsiveness observed in the DF (**Figure 4C**).

These results extend previous observations showing stepwise increases in glucose uptake, mitochondrial mass, and respiratory reserve across CXCR4/CD5-defined CLL fractions (11), and demonstrate that CENCAT can resolve functional metabolic dependencies within these intraclonal populations. More importantly, reveal that early-onset CLL exhibits an enhanced responsiveness to TME-derived signals, particularly CD40L/IL-4 stimulation, characterized by glucose dependence fitness and greater capacity for metabolic reprogramming.

The metabolic heterogeneity observed across intraclonal fractions in early-onset CLL is also consistent with previous reports linking glycolytic capacity and glycolytic reserve to IGHV mutational status and disease progression (44). Given that unmutated CLL cells display increased glycolytic activity and are generally associated with a more aggressive clinical course, the similar metabolic phenotype identified in early-onset CLL suggests that enhanced glucose utilization may represent a biological mechanism contributing to accelerated disease progression in this subgroup.

Aging profoundly influences immune competence through the process of immunosenescence (45, 46). Therefore, it is plausible that age at disease onset may affect the interplay between leukemic cells and the immune microenvironment, in CLL. However, despite evaluating multiple markers associated with T-cell exhaustion in CLL (47), no significant differences were observed between younger and older patients. These findings suggest that the biological differences associated with age at disease onset are more likely driven by intrinsic leukemic cell properties than by major alterations in T-cell exhaustion.

The observation that young- and older-patients exhibit remarkably similar T-cell exhaustion and memory profiles may be explained by the profound impact of CLL on immune aging. CLL has been proposed to act as a potent accelerator of immunosenescence, whereby chronic exposure to leukemic antigens drives the expansion of antigen-experienced CD8^+^ T cells and promotes the progressive accumulation of differentiated and exhausted populations (48). This process generates a “prematurely aged” immune phenotype that closely resembles physiological aging, characterized by contraction of the naïve T-cell compartment and expansion of memory subsets (49, 50).

Consistent with this concept, our analysis of CD8^+^ T-cell subsets revealed a largely conserved distribution of memory and exhaustion populations across younger- and older-onset CLL patients. This apparent convergence likely reflects the dominant influence of chronic antigenic stimulation imposed by the leukemic clone, which may override many of the immunological differences typically associated with chronological aging. Nevertheless, younger patients retained a larger naïve CD8^+^ T-cell compartment, in agreement with the well-established inverse relationship between age and naïve T-cell abundance (51). The preservation of this compartment is also consistent with longitudinal studies in CLL mouse models demonstrating a progressive decline in naïve CD8^+^ T cells as disease burden and chronic antigen exposure increase over time (49).

Taken together, these findings support the notion that the CLL microenvironment drives profound T-cell differentiation and exhaustion regardless of patient age, pushing the immune system of younger patients toward a functional and phenotypic state that resembles that of substantially older individuals. Thus, while chronological age influences the size of the residual naïve T-cell pool, the overall architecture of the T-cell compartment appears to be shaped predominantly by disease-driven immunosenescence.

In summary, our study validates in an independent Uruguayan cohort the distinctive clinical behavior of early-onset CLL, characterized by a shorter time to first treatment despite prolonged overall survival.

By applying the CENCAT methodology to investigate metabolic reprogramming following TME stimulation, we demonstrate that CLL cells from younger patients, particularly those within the proliferative fraction, exhibit enhanced metabolic fitness characterized by increased reliance on glucose metabolism rather than mitochondrial dependence.

Given that elevated glycolytic activity is a hallmark of progressive and unmutated CLL and has been associated with adverse clinical outcomes, the metabolic phenotype identified in early-onset CLL suggests that enhanced glucose utilization may constitute a biological mechanism underlying its accelerated disease course. These findings provide new insights into the biology of early-onset CLL and highlight metabolic reprogramming as a potential contributor to disease progression and a candidate therapeutic vulnerability in this clinically relevant subgroup.

## Data Availability

The original contributions presented in the study are included in the article/**Supplementary Material**. Further inquiries can be directed to the corresponding authors.
