# Supplementary material for: Early-onset CLL is characterized by enhanced microenvironment-driven metabolic fitness, increased proliferative bias, and accelerated disease progression
Source: Front Immunol. 2026 Sep 7;17:1936737. doi: 10.3389/fimmu.2026.1936737 (PMC13590693; doi:10.3389/fimmu.2026.1936737)
Supplement: Supplementary file 1 [file DataSheet1.pdf]

Supplementary Figure 1

ID	Age	Sex	Binet Stage	Mutational Status	Limphocitosis	FISH	Stereotipe	Metabolic studies	Proliferative fraction	T-cell exhaustion
CLL-01	71	F	A	Mutated	17.400	del_13q14	unassigned			X
CLL-02	73	M	A	Mutated	12.600	del_13q14	unassigned			X
CLL-03	72	F	A	Mutated	22.210	tris_12	CLL#4	X		
CLL-04	81	M	B	Unmutated	129.940	tris_12	unassigned	X		
CLL-05	77	M	C	Unmutated	7.178	del_17p	unassigned	X		X
CLL-06	75	M	A	Mutated	27.840	normal	unassigned	X		X
CLL-07	76	M	A	Unmutated	47.000	del(11q)	unassigned	X		
CLL-08	85	M	B	Unmutated	100.000	normal	unassigned	X		
CLL-09	63	M	B	Unmutated	237.500	del_13q14	unassigned	X		
CLL-10	66	F	A	Mutated	19.450	normal	CLL#4		X	X
CLL-11	72	M	A	Unmutated	58.600	normal	unassigned	X	X	X
CLL-12	65	M	A	Unmutated	42.500	del_13q14	unassigned	X	X	X
CLL-13	79	F	B	Mutated	81.000	normal	unassigned		X	X
CLL-14	73	F	C	Mutated	200.000	del_13q14	CLL#64B		X	X
CLL-15	74	F	A	Mutated	35.000	del_13q14	unassigned		X	X
CLL-16	79	M	A	Unmutated	26.397	tris_12	CLL#1		X	X
CLL-17	73	F	B	Mutated	54.170	normal	unassigned		X	
CLL-18	67	M	C	Unmutated	5.400	del(11q)	CLL#6		X	
CLL-19	70	M	A	Mutated	40.220	tris_12	unassigned		X	X
CLL-20	73	F	C	Mutated	30.650	del_17p	CLL#7H		X	X
CLL-21	67	F	C	Unmutated	80.000	del_13q14	CLL#3		X	
CLL-22	77	M	A	Unmutated	23.400	del(11q)	CLL#3		X	X
CLL-23	70	M	A	Mutated	18.800	del_13q14	unassigned		X	X
CLL-24	72	F	B	Unmutated	21.100	tris_12	unassigned		X	
CLL-25	47	F	A	Mutated	48.700	del_13q14	CLL#4	X		X
CLL-26	45	M	A	Mutated	19.500	del_13q14	unassigned	X		X
CLL-27	42	M	A	Mutated	11.006	normal	unassigned	X		X
CLL-28	49	M	B	Unmutated	45.000	normal	CLL#7H	X	X	X
CLL-29	50	M	C	Mutated	143.630	tris_12	unassigned	X	X	X
CLL-30	49	F	C	Mutated	118.000	del_13q14	unassigned	X		X
CLL-31	54	M	C	Unmutated	17.900	normal	unassigned	X	X	X
CLL-32	46	M	A	Mutated	48.390	normal	unassigned	X		X
CLL-33	33	M	C	Unmutated	459.900	del_13q14	unassigned	X		X
CLL-34	44	M	B	Unmutated	183.000	normal	unassigned	X	X	
CLL-35	53	M	A	Unmutated	39.100	del(11q)	unassigned		X	X
CLL-36	53	F	A	Unmutated	225.000	del_17p	CLL#28A		X	
CLL-37	50	M	A	Unmutated	21.600	del(11q)	unassigned		X	X
CLL-38	52	M	B	Unmutated	138.940	del_13q14	CLL#12		X	
CLL-39	48	F	B	Unmutated	25.200	del_13q14	unassigned		X	X
CLL-40	52	M	C	Unmutated	421.000	tris_12	CLL#5		X	
CLL-41	54	F	B	Mutated	25.600	del_13q14	unassigned		X	X
CLL-42	55	F	B	Unmutated	75.700	del(11q)	unassigned		X	
CLL-43	45	M	B	Unmutated	93.500	del_13q14	CLL#5		X	
CLL-44	55	M	A	Unmutated	30.000	del_13q14	CLL#6		X	X
CLL-45	55	M	C	Mutated	40.000	normal	unassigned		X	X
